# Musculoskeletal Disorders and Perceived Work Demands among Female Nurses at a Tertiary Care Hospital in India

**DOI:** 10.1155/2016/5038381

**Published:** 2016-07-14

**Authors:** Apexa S. Raithatha, Daxa G. Mishra

**Affiliations:** KM Patel Institute of Physiotherapy, Shree Krishna Hospital, Gokal Nagar, Karamsad, Gujarat 388325, India

## Abstract

*Introduction*. Musculoskeletal disorders (MSD) are common among nurses and can affect patient outcomes. There is a dearth of literature on MSD among Indian nurses. The study objective was to measure prevalence of MSD and their association with perceived work demands and sociodemographic variables among female nurses at a tertiary care hospital in rural India.* Methods*. A cross-sectional study was undertaken in 2013 through interviewer administered questionnaires which comprised three parts: sociodemographic data, modified Nordic questionnaire, and perceived physical and psychological work demands.* Results*. 296 nurses with a mean age of 30.4 years participated. Prevalence of any MSD in the last seven days was 60.5% with low back pain being the most common and elbow pain the least common. Occurrence of any MSD was associated with age, number of children, working hours at home, BMI, and total work experience. High perceived physical demands score was associated with lower back (OR: 3.06) and knee pain (OR: 7.73).* Conclusion*. Prevalence of MSD was high and occurrence of lower back and knee MSD was associated with perceived physical demands. This information should be used as a benchmark and guiding tool for designing work place interventions to improve working conditions and health of nurses.

## 1. Introduction

“Musculoskeletal disorders” (MSD) include a wide range of inflammatory and degenerative conditions affecting the muscles, tendons, ligaments, joints, peripheral nerves, and supporting blood vessels [1]. Work related MSD by definition are a subset of musculoskeletal disorders that arise from occupational exposures [2]. They are common among health care workers. The nursing population, that constitutes about 33% of the hospital workforce accounts for 60% of these MSD [3]. The job requirements of nursing personnel associated with providing medications to patients, maintaining hygiene of patients, looking after the dietary needs, and so forth predispose them to a very high risk for development of MSD.

The most commonly reported biomechanical risk factors for MSD include excessive repetition, awkward postures, and heavy lifting [4]. Nurses often conduct patient handling by bending their waist and maintaining an uncomfortable posture towards the opposite side of the bed or chair, increasing the risk of back pain [5]. Shift work may be a demanding situation because it raises problems for restoring work and no work activities. Shift schedules that involve night duties also disturb circadian rhythm and put different workload demands and reduce adequate communication and participation in preventive activities than the other work schedules [6]. MSD have a significant impact on the quality of life [1]. They contribute to lost work time or absenteeism and reduced work participation and quality of work output, resulting in a considerable economic burden on the individual, the organization, and the society as a whole [3, 5].

Several authors have reported the prevalence of MSD among nurses in the developed populations worldwide [3, 7–9]. However, data on prevalence of MSD are limited in India. The overburdened health care system of India due to the lack of adequate and skilled medical and paramedical personnel puts additional demands on its human resources. Moreover majority of the nursing staff in India are females who have added responsibilities of looking after their families which increases the physical and psychological burden further. In such a scenario, the Indian nurses appear to be at a higher risk of development of MSD as compared to their western counterparts. In order to gain an understanding about the prevalence of MSD and its associated risk factors, this study was undertaken at a rural tertiary care hospital in India. The findings of the study can be utilised to plan and evaluate work place interventions to reduce the burden of MSD among nurses.

## 2. Materials and Methods

A cross-sectional descriptive study was undertaken at a tertiary care hospital located in the Anand district of the Gujarat state located in the western part of India. Ethical clearance was obtained from the institutional Human Research Ethics Committee. The inclusion criteria were female nurses working at the hospital for at least one year. Nurses reporting as having congenital deformities, traumatic conditions, neurological conditions, and gynaecological conditions were excluded from the study. The data was collected by administering a structured, coded, and pretested questionnaire on a one-to-one basis in local language. The questionnaire consisted of mainly three divisions, namely, demographic data, Nordic questionnaire, and perceived physical and psychological demand items. The Nordic questionnaire has been validated to screen musculoskeletal disorders in different populations [10, 11]. Additional questions on frequency of MSD were added to Nordic's questionnaire to obtain better information regarding musculoskeletal problems. For the purpose of analysis the outcome variable for presence of MSD was calculated on the basis of frequencies. All those having a frequency of “often” or “almost daily” were considered as having high frequency MSD. Self-perception of their work conditions was measured by using a structured and coded questionnaire based on the study by [8] and the Job Content Questionnaire (JCQ) [12]. Physical and psychological demand scores were calculated based on responses to different questions. For physical items, responses were dichotomised (1 and 2 versus 3 and 4) and then summed up to produce a total score which ranged from 0 to 12. Thereafter the scores were classified into three categories, as low (0 to 3), medium (3 to 9), and high (10 to 12). Items for perceived psychological demands were also dichotomised and summed up to produce a total score which ranged from 0 to 8. The scores were then categorised as low (0 to 5) and high (6 to 8) [8].

Data entry was done using Microsoft Excel 2007 and data analysis was done with the SPSS version 15.0. Means, 95% Confidence Intervals (CI), frequencies, and percentages were used for descriptive analysis. Cronbach's alpha was calculated to measure internal consistency of various items for the physical demand score and psychological demand score. For univariate analysis, the normality of numerical variables was tested using the Kolmogorov-Smirnov test. To test the association between numerical independent variables and occurrence of MSD, Mann-Whitney* U* test was applied as the variables were not normally distributed [13]. Chi-square (*χ* ^2^) test was used for testing associations between categorical variables. Association of MSD with individual items of perceived physical and psychological demands was measured using Multiple Binary Logistic Regression analysis.

## 3. Results

There were a total of 320 nurses eligible for the study out of which 296 participated in the study (response rate: 92.5%).  Table 1 describes the sociodemographic and work profile of the participants. Mean age was 30.44 years (95% CI: 29.46–31.42) with majority of the participants in the age group of 20–25 years (44.9%). Mean duration of work done by nurses at home was 3.81 hours per day. 248 (83.8%) nurses were having shifting duties and total duration of work in a week was 48 hours for each of them. The mean duration of total work experience was 7.96 years and that at the current hospital was 7 years. Among the participants, majority (89.5%) were staff nurses. The mean BMI was 21.58 kg/m^2^ (95% CI: 21.14–22.02) and 19.3% and 16.2% were underweight and obese, respectively.

The prevalence of different MSD is shown in Table 2. Prevalence of any MSD in the last 12 months and past 7 days was 89.2% and 60.5%, respectively. Low back pain was the most commonly reported MSD whereas elbow pain was the least commonly reported.  Table 3 shows association of any MSD with important sociodemographic variables tested through univariate analysis. The mean age of the participants, mean number of children at home, mean duration of working hours at home, mean duration of work experience at the current hospital, and mean duration of total work experience were significantly higher in those having any MSD as compared to those not having any MSD. Moreover the prevalence of any MSD was higher in those having a housemaid at home and married nurses as compared to those not having a housemaid and those who were unmarried, respectively. The difference in the prevalence of MSD across different categories of the nursing staff position, posting in different areas, and the type of duty shifts was not significant.

Table 4 shows association of various items related to physical demands at work and psychological demands at work with the occurrence of any MSD tested through Multiple Binary Logistic Regression. Among all the items, the physical demand perception “in my job, I am working for long periods with my body in awkward positions” was significantly associated with the occurrence of any MSD. Cronbach's alpha for the physical and psychological work demands items was found to be 0.833 and 0.762, respectively. Thereafter a logistic regression analysis (Table 5) was done with the total physical demands score, total psychological demands score, age, BMI, work experience, number of children < 5 years of age, duration of work at home, and having a housemaid as independent variables and occurrence of different MSD as the dependent variable. It was found that a high score for perceived physical demands was significantly associated with the occurrence of MSD in the lower back and knee regions, presence of housemaid at home with MSD in neck and lower back, and presence of children with upper and lower back regions.

## 4. Discussion

This was a cross-sectional study carried out at a tertiary care hospital in Gujarat, India, to find the prevalence of musculoskeletal disorders and its association with self-perceived work demands and other sociodemographic and work related variables among the female nursing staff.

The prevalence of MSD in any body part was 89.2% in the last 12 months and 60.5% in the past 7 days and 59.5% of the nurses reported occurrence of MSD with high frequency, that is, MSD occurring “often” or “almost daily.” The prevalence of MSD in the last 12 months in the lower back region (69.6%) was the highest followed by the same in the neck (34.5%), upper back (29.1%), ankle/feet (27.0%), and knee (26.4%) regions in the last 12-month period. This finding is consistent with the findings obtained from several other studies [3, 7, 9, 14, 15]. The prevalence of MSD in the elbow region was the least. Similar finding was also obtained in the study conducted by [7] and in a study conducted in rural Japan nurses [16]. The mean age of the participants with MSD in any body part was higher than that of those without MSD and the difference was statistically significant. Similar findings were also observed in the study undertaken on Nigerian nurses by Tinubu et al. [3]. It is a well-known fact that as the age increases, functional capacity of an individual decreases resulting in a higher incidence of MSD [17].

The mean number of children (*p* < 0.001) and mean number of domestic work hours' duration (*p* = 0.001) were higher in the group having any MSD as compared to those not having any MSD which could be because of the fact that the greater amount of workload and responsibility at home leads to higher prevalence of MSD. Marital status was significantly associated (*p* < 0.001) with any MSD, which could be due to the fact that, in Indian culture, after marriage the responsibility increases and so does the domestic work, thus increasing the physical demands of work. Those having a housemaid were having a higher prevalence of any MSD as compared to those not having the housemaid. Similar findings were obtained for the prevalence of low back pain. The duration of domestic work was also associated with increased odds of MSD over lower back region which reflects the effect of higher domestic work demands on the occurrence of MSD over lower back region. It has been known that the double work burden or the simultaneous engagement in two different types of tasks, namely, paid job and household work, increases psychological and physical demands from an individual resulting in an increased risk of MSD [18]. On doing further analysis it was found that as the frequency of the back pain increased the proportion of nurses having a housemaid increased sequentially. It reflects that the higher frequency of lower back pain might be leading to appointment of a house maid for doing routine household work. It can be explained by the concept of reverse causality [19].

A significant difference was not found in the prevalence of any MSD in those having rotating or fixed duty shifts. It could be due to the fact that the shift duties are worked out in such a way that no one is required to do more than two consecutive shifts (i.e., morning, evening, and night) and the shifts are arranged in forward manner (i.e., morning, evening, and night) which is known to reduce side effects like fatigue. This is a well-known strategy to reduce the burden of rotating duties [20]. The mean work experience in years was significantly higher in those having MSD in any body part as compared to those not having the same. Similar findings were also seen in the study by Tinubu et al. [3]. As worker's seniority and job experience increase they tend to have fixed shift, so fixed shift is associated with increased years of employment and increased age which was also seen in our study [21]. It was found that the odds of development of any MSD were significantly higher (2.723 (95% CI: 1.336–5.549)) among those who perceived that they had to work for long periods with the body in awkward positions. This finding was further supported by other studies as well [8, 22, 23].

The findings of the study should be generalised with caution. The variability of the workload and ergonomics knowledge of the study respondents in different areas of the hospital may influence homogeneity. In a cross-sectional study survivor effects will typically decrease the observed associations between symptomatic disorders and physically demanding jobs [24]. Since the analysis was limited to currently working nurses, we may have excluded nurses who had left jobs due to MSD or other health conditions during the study time. The MSD reported here may be work related or due to other factors and it is very difficult to distinguish the two [25]. Furthermore, the cross-sectional design prevented us from drawing conclusions on the temporal order of relationships. Further studies may be undertaken with posture analysis systems to get an in-depth understanding of the issues. Detailed ergonomic evaluation of the work place should be done to thorough observations and other techniques to get important clues related to the problem.

## 5. Conclusions

The study reported a high prevalence of MSD and occurrence of lower back and knee MSD was associated with perceived physical demands among nurses in a rural based tertiary care medical college hospital. The findings of such a study can be utilised in the development of a work place intervention addressing the risk factors and preventive measures for the MSD identified. The findings can serve as a benchmark for evaluation of the effect of the intervention.

## Figures and Tables

**Table 1:** Descriptive analysis: sociodemographic and work related information.

Variable	Value
Mean age (95% CI)	30.44 (29.46–31.42)

Mean number of family members (95% CI)	5.13 (4.88–5.39)

Mean number of children (95% CI)	0.65 (0.55–0.75)

Family type (percent)	
Nuclear family	187 (63.2%)
Joint family	109 (36.8%)

Marital status (percent)	
Unmarried	133 (44.9%)
Married	162 (54.7%)
Others	1 (0.4%)

Appointment of housemaid at home (percent)	
Yes	60 (20.3%)
No	236 (79.7%)

Mean duration of domestic work in hours (95% CI)	3.81 (3.6–4.03)

Clinical posting (percent)	
OPD	19 (6.4%)
Ward	119 (40.2%)
OT	32 (10.8%)
ICU	110 (37.2%)
Emergency care unit	16 (5.4%)

Type of shifts	
Rotatory	248 (83.8%)
Fixed	48 (16.2%)

Mean years of work experience (95% CI)	7.96 (7.10–8.81)

(OPD: outpatient department, OT: operation theater, and ICU: intensive care unit).

**Table 2:** Prevalence of different types of MSD over the last 12 months and last 7 days and with high frequency.

MSD	Prevalence (last 12 month)^a^	Prevalence (last 7 days)^b^	Prevalence with high frequency^*∗*^
Any MSD	264 (89.2%)	179 (60.5%)	176 (59.5%)
Neck	102 (34.5%)	48 (16.2%)	36 (12.2%)
Shoulder	62 (20.9%)	26 (8.8%)	22 (7.5%)
Elbow	4 (1.4%)	2 (0.7%)	1 (0.3%)
Wrist/hands	55 (18.6%)	24 (8.1%)	15 (5.1%)
Upper back	86 (29.1%)	46 (15.5%)	35 (12.0%)
Lower back	206 (69.6%)	120 (40.5%)	108 (37.0%)
Thighs/hips	14 (4.7%)	10 (3.4%)	9 (3.1%)
Knees	78 (26.4%)	35 (11.8%)	37 (12.7%)
Ankle/foot	80 (27.0%)	38 (12.8%)	34 (11.6%)

Note: ^a^occurrence of MSD in the last 12 months.

^b^Occurrence of MSD in the last 7 days.

^*∗*^Prevalence with high frequency: if the participant responded as having the MSD “often” or “almost daily”, it was considered as high frequency.

MSD: musculoskeletal disorders.

**Table 3:** Association of MSD with various sociodemographic variables (univariate analysis).

Variable	Any MSD		*p* value
	Yes	No	
Mean age (95% CI)	31.76	28.63	0.01^a^

Family type			
Nuclear family	110 (59.8%)	77 (40.2%)	0.842^b^
Joint family	64 (59.3%)	45 (40.7%)	

Number of family members			
≤5 members	120 (58.8%)	88 (40.2%)	0.739^b^
>5 members	53 (60.9%)	35 (30.1%)	

Mean number of children (95% CI)	0.8333	0.3814	<0.001^a^

Duration of working hours at home	4.1322	3.3602	0.001^a^

Appointment of housemaid at home			
Yes	45 (75.00%)	15 (25.00%)	0.006^b^
No	129 (55.6%)	107 (44.4%)	

Marital status			
Unmarried	62 (47.3%)	71 (52.7%)	<0.001^b^
Married	111 (69.4%)	51 (30.6%)	

^a^Mann-Whitney *U* test.

^b^Pearson Chi-square test.

A *p* value of <0.05 was considered as significant and it has been made bold wherever it was found significant.

**Table 4:** Association of occurrence of any MSD with perceived physical and psychological demands at work, Multiple Logistic Regression analysis.

Perceived physical and psychological demand items	OR for any MSD (95% CI)
*Perceived physical demand items*	
My job requires lots of physical efforts	1.383 (0.744–2.573)
My job requires rapid and continuous physical activity	1.215 (0.613–2.407)
In my job, I am often moving/lifting very heavy loads	0.746 (0.414–1.342)
In my job, I am working for long periods with my head or arms in awkward positions	0.731 (0.359–1.489)
In my job, I am working for long periods with my body in awkward positions	**2.723 (1.336–5.549)**
Lift or lower patients/objects to/from floor	0.817 (0.438–1.523)
Lift or lower objects to/from shoulder height	1.820 (0.912–3.629)
Work while bent or twisted at waist	1.575 (0.908–2.731)
Push/pull heavy objects or people	1.427 (0.740–2.752)
Stand in one place/static position (>30 minutes)	0.648 (0.366–1.148)
Perform repetitive motions with hands/wrists	1.023 (0.574–1.823)
Apply pressure with hands/fingers (e.g., to prevent bleeding)	1.274 (0.711–2.282)

*Perceived psychological demand items *	
My job requires working very hard	0.952 (0.480–1.889)
My job requires working very fast	1.113 (0.443–2.798)
My job requires an excessive amount of work	1.275 (0.670–2.427)
My job requires long periods of intense concentration on the task	1.736 (0.859–3.508)
My job requires enough time to get the job done	1.095 (0.639–1.875)
My job is free from conflicting demands that others make	0.928 (0.515–1.673)
My job requires waiting on work from other people or departments	1.288 (0.727–2.281)
My job has tasks that are often interrupted before they can be completed	1.155 (0.654–2.039)

**Table 5:** Association of different MSD with total physical demands score, total psychological demands score, and selected sociodemographic and work related variables (Multiple Logistic Regression analysis with each MSD).

Independent variables	Neck	Upper back	Lower back	Knee	Ankle
Age	1.088 (0.945–1.252)	0.829 (0.668–1.028)	0.946 (0.846–1.058)	1.073 (0.945–1.218)	0.996 (0.859–1.156)

BMI	0.927 (0.826–1.040)	0.981 (0.876–1.098)	0.981 (0.907–1.060)	1.065 (0.957–1.184)	0.996 (0.892–1.112)

Work experience	0.968 (0.818–1.145)	1.184 (0.943–1.488)	1.034 (0.911–1.174)	0.985 (0.848–1.144)	1.025 (0.865–1.214)

Number of children under 5 years of age	2.002 (0.747–5.362)	**3.604 (1.387–9.363)**	**2.202 (1.044–4.646)**	0.967 (0.322–2.904)	1.028 (0.360–2.935)

Duration of work at home	1.049 (0.840–1.310)	0.870 (0.681–1.110)	**1.228 (1.046–1.440)**	1.107 (0.882–1.388)	1.129 (0.899–1.418)

Having a housemaid at home	**2.935 (1.306–6.596)**	1.378 (0.579–3.277)	**2.723 (1.470–5.045)**	0.371 (0.120–1.148)	1.159 (0.480–2.800)

Physical demands					
Low	1	1	1	1	1
Medium	0.841 (0.254–2.790)	1.117 (0.304–4.103)	1.987 (0.846–4.668)	1.980 (0.577–6.793)	1.062 (0.892–1.792)
High	2.255 (0.530–9.586)	1.839 (0.382–8.856)	**3.061 (1.005–9.325)**	**7.726 (1.716–34.785)**	1.890 (0.901–2.100)

Psychological demands	1.873 (0.828–4.238)	1.106 (0.506–2.417)	0.994 (0.579–1.706)	0.757 (0.332–1.722)	0.870 (0.398–1.900)
